# Reprogramming of Cellular Metabolism and Its Therapeutic Applications in Thyroid Cancer

**DOI:** 10.3390/metabo12121214

**Published:** 2022-12-03

**Authors:** Yuji Nagayama, Koichiro Hamada

**Affiliations:** 1 Department of Molecular Medicine, Atomic Bomb Disease Institute, Nagasaki University, 1-12-4 Sakamoto, Nagasaki 852-8523, Japan; 2 Department of General Medicine, Nagasaki University Graduate School of Biomedical Sciences, Nagasaki 852-8501, Japan

**Keywords:** thyroid cancer, metabolomics, the Warburg effect, glycolysis, TCA cycle, electron transport chain, lipid metabolism, glutaminolysis

## Abstract

Metabolism is a series of life-sustaining chemical reactions in organisms, providing energy required for cellular processes and building blocks for cellular constituents of proteins, lipids, carbohydrates and nucleic acids. Cancer cells frequently reprogram their metabolic behaviors to adapt their rapid proliferation and altered tumor microenvironments. Not only aerobic glycolysis (also termed the Warburg effect) but also altered mitochondrial metabolism, amino acid metabolism and lipid metabolism play important roles for cancer growth and aggressiveness. Thus, the mechanistic elucidation of these metabolic changes is invaluable for understanding the pathogenesis of cancers and developing novel metabolism-targeted therapies. In this review article, we first provide an overview of essential metabolic mechanisms, and then summarize the recent findings of metabolic reprogramming and the recent reports of metabolism-targeted therapies for thyroid cancer.

## 1. Introduction

Thyroid cancer is the most common endocrine malignancy, whose incidence rate has rapidly increased during the last several decades [1]. Thyroid cancers derived from thyroid follicular epithelial cells can be divided into two subgroups: differentiated [papillary (PTC) and follicular (FTC)] and de-differentiated [poorly differentiated and anaplastic (ATC)]. PTC is the most common type, in which the point mutation in BRAF (BRAF^V600E^) has been shown to be the most frequent driver mutation, followed by RET/PTC (the chromosomal rearrangement) and the point mutation in RAS, all of which stimulate the RAS-RAF-MEK-ERK signaling pathway [2]. Oncocytic thyroid (Hürthle cell) cancer is a unique type of thyroid malignancy characterized by enlarged eosinophilic cytoplasm and accumulation of dysfunctional mitochondria [3,4], in which impaired mitochondrial function is attributed to the loss-of-function mutations in mitochondrial DNA encoding for the complex I or III of the electron transport chain (ETC) [5]. 

Surgery, radioactive iodine (RAI) treatment with ^131^I and thyrotropin suppression therapy with excess L-thyroxine have long been choices of treatment modalities in most thyroid cancer patients, and have gained high disease remission; however, recurrence sometimes occurs in a subgroup of patients in whom RAI resistance has developed, mainly because of dedifferentiation. Targeting therapy, such as the use of tyrosine kinase inhibitors, has recently been introduced as a novel therapeutic choice with clear therapeutic benefits; however, at the same time, some adverse effects and/or intrinsic/acquired resistance have been observed. Thus, development and introduction of a novel therapeutic approach is urgently necessary. Targeting metabolic reprogramming may have potential for this purpose.

Metabolism is a series of life-sustaining chemical reactions in organisms, which produces the energy required for cellular processes and building blocks for cellular constituents of proteins, lipids, carbohydrates and nucleic acids. Cancer cells frequently reprogram their metabolic behaviors to adapt their own rapid proliferative status and altered—i.e., nutrient-limiting and/or hypoxic—tumor microenvironments. Thus, metabolic reprogramming is a hallmark of cancers [6]. All the constituents can be subjected to metabolic reprogramming at different levels in distinct cancers and/or even in different parts of a cancer. The most famous is “the Warburg effect”, that is, enhanced glycolysis even in oxygen-sufficient conditions [7]. Moreover, altered metabolisms of amino acids including glutamine and serine/glycine (connected to the one-carbon metabolism), fatty acids and redox equilibrium are also executed. These alterations are at least in part attributed to genetic mutations. Elucidation of the mechanism(s) for these metabolic reprogramming is expected to uncover the molecular events of malignant process and facilitate the development of novel diagnostic and therapeutic modalities. Thus, the aim of this review is to summarize the recent findings of metabolic reprogramming in thyroid cancers and discuss the therapeutic potentials targeting this phenomenon. Although altered metabolic status has been evaluated with not only tissues or cells but also body fluids, such as serum/plasma, urine, etc. [5,8], here we focus on the data obtained with tissue and cell samples, because these data best reflect metabolic reprogramming within cancer cells, and the etiology of metabolite alterations in body fluids is sometimes unclear. Diagnostic potential of omics including metabolomics has recently been published elsewhere [9]. 

## 2. Glucose Metabolism

### 2.1. Physiology

**Figure 1:**
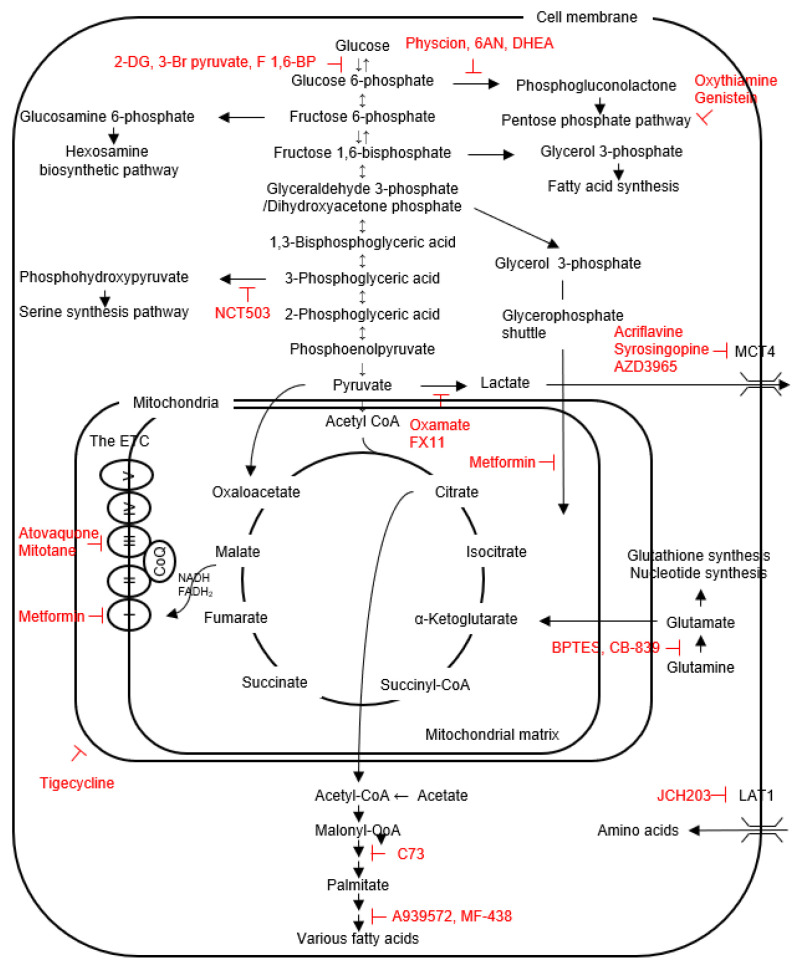
The scheme for the intracellular metabolic pathways and inhibitors (shown in red) of cancer metabolism, mainly targeting membrane transporters and enzymes.

Glycolysis is one of the main metabolic pathways, where one molecule of glucose is converted into two molecules of pyruvate with the concurrent generation of two molecules of ATP [called the cytoplasmic substrate level phosphorylation (cSLP)] [10] (Figure 1). In the physiological condition where enough oxygen is present, pyruvate normally enters the tricarboxylic acid (TCA) cycle within mitochondria to produce NADH and FADH_2_ and two molecules of ATP or GTP per glucose [called the mitochondrial SLP (mtSLP)] [10,11]. NADH and FADH_2_, reducing agents, are subsequently used to generate an additional 34 molecules of ATP per glucose in the ETC. This ATP-producing reaction in the ETC is called oxidative phosphorylation (OXPHOS). However, when oxygen is insufficient, or in the case of the metabolic reprogramming frequently observed in cancers (the Warburg effect; see below), pyruvate is converted into lactate in the cytosol by lactate dehydrogenase (LDH) with concurrent regeneration of NAD^+^ from NADH. In this case, ATP production in mitochondria (the mtSLP and the ETC) is blocked, thereby generating less ATP, but increased NAD^+^ drives the first step of glycolysis flux and increases glycose uptake. In either case, glucose metabolism starts with translocation of glucose across the plasma membrane through glucose transporters (GLUTs), and is accomplished by a series of reactions catalyzed with several enzymes including hexokinase (HK), phosphoglucose isomerase, etc. in the glycolysis pathway, pyruvate dehydrogenase (PDH), citrate synthase, etc. in the TCA cycle, and NADPH dehydrogenase, succinate dehydrogenase, etc. in the ETC. 

In addition to ATP generation, glycolysis also supplies biosynthetic intermediates for cell proliferation and survival through the several branching pathways. The first example is glucose-6-phosphate (G6P), the first metabolic intermediate in the glycolysis pathway, which can be diverted into the pentose phosphate pathway (PPP) to drive NADPH generation to reduce reactive oxygen species (ROS) produced mainly by the ETC (an oxidative branch), and to supply ribose 5-phosphate for nucleotide biosynthesis (a non-oxidative branch); the second is fructose 6-phosphate used for the hexosamine biosynthetic pathway (HBP) generating uridine diphosphate-*N*-acetylglucosamine (UDP-GlcNAc), a substrate for protein *N*- and *O*-glycosylation reaction [12]; the third is fructose 1,6-bisphosphate (F1,6-BP) used for synthesis of glycerol 3-phosphate required for triacylglycerol formation [12]; the forth is 3-phosphoglycerate (3PG) used for the serine/glycine metabolism and one carbon metabolism, which support diverse cellular processes, including methylation, antioxidant defense and nucleotide metabolism [12]; the fifth is pyruvate used to generate oxaloacetate by pyruvate carboxylase (PCB) as an anaplerotic flux into the TCA cycle [13]. 

### 2.2. Glucose Metabolism Reprogramming in Thyroid Cancers

Warburg first observed that proliferating cancer cells augment glycolysis—the conversion of pyruvate to lactate, not to acetyl-CoA—even in the presence of enough oxygen [7,14]. This metabolic shift, called the Warburg effect or aerobic glycolysis, is insufficient for ATP production but is advantageous for providing additional biosynthetic precursors to support rapid cell proliferation (see above), for increasing survival capacity in fluctuating oxygen levels within a tumor and for acidifying the tumor microenvironments by releasing lactate through the monocarboxylate transporter 4 (MCT4), that augments migration/invasion of cancer cells and inhibits anti-cancer immune response [15,16]. To compensate lower ATP production, glucose uptake is generally increased in glycolytic cancer cells. Although aerobic glycolysis was originally thought to be attributed to impaired function of mitochondria, it is now well recognized that the function of mitochondria is largely intact [17]. Thus, there are now several potential reasons for the shift from OXPHOS to aerobic glycolysis [18]: (i) suppression of OXPHOS and/or upregulation of glycolysis by the activation of oncogenes or inactivation of tumor suppressors (such as MYC and TP53); (ii) the triggering of a strong hypoxic response by activation of hypoxia-inducible factor 1α (HIF1α) that turns down oxygen-dependent respiration (by upregulation of pyruvate dehydrogenase kinase and thereby inhibiting PDH) and increases glycolytic activity (by increasing GLUT1 and downstream glycolytic enzymes such as HK2, pyruvate kinase isoform M2 (PKM2; an embryonic splice variant of PK) and LDHA (see below)); (iii) the redirection of glucose catabolism towards macromolecular biosynthesis; (iv) mitochondrial dysfunction, all of which force cells to rely on glycolysis, but (iv) is rare.

In human non-oncocytic thyroid cancers, clinical data show higher concentrations of lactate [19,20,21,22,23,24,25] and higher expression levels of the glycolysis-related molecules, such as GLUT1, HK2, LDHA and MCT1 and 4 (these isotypes are frequently over-expressed in cancers), than normal counterparts [26,27,28,29,30,31]; these are especially evident in cancers from aged patients [32]. The detailed characteristics of individual enzymes and transporters in the glycolytic pathway mentioned above have recently been described [33,34]. Expression levels of glycolysis-related molecules are in general correlated with aggressiveness and poor prognosis [35,36]. For example, (i) GLUT1 expression is higher in ATCs versus (vs.) PTCs, and in FTCs vs. follicular adenomas [35], and is positively correlated with dedifferentiation status, aggressiveness and proliferation, and negatively correlated with sodium iodine symporter (NIS) expression, a differentiation marker [37]. (ii) Glucose uptake estimated with positron emission tomography scanning with ^18^F-fluorodeoxyglucose (FDG-PET) is negatively correlated with differentiation levels [38], and thus has a prognostic value [39]. (iii) Expression of PKM2 is higher in thyroid cancers compared to normal/hyperplasia/benign tumors and is associated with tumor aggressiveness and poor prognosis [40,41,42]. (iv) LDHA expression is also positively correlated with aggressiveness and negatively with overall survival and progression-free interval [31,43]. 

Regarding the correlation between glycolytic status and gene mutations, BRAF^V600E^, the most frequent driver mutation in thyroid cancers, is reported to suppress OXPHOS and induce glycolysis. Thus, glucose uptake in FDG-PET and expression of GLUT1/GLUT3 are higher in PTCs with BRAF^V600E^ than those without [44,45,46,47]. The similar effect of the mutant RAS has also been described in other cancers [34]. 

Expression of glucose-6-phosphate dehydrogenase (G6PD), an enzyme that mediates G6P to 6-phosphogluconolactone in the oxidative branch of the PPP, and transketolase, an enzyme that mediates the non-oxidative branch of the PPP, is higher in PTCs vs. normal tissues [48,49], as is 6-phosphogluconate dehydrogenase, another enzyme in the oxidative branch, in tall cell variants vs. classical PTCs [50], suggesting higher activities of the PPP shunt in the formers. Thus, deviations of glycolytic metabolic intermediates to the PPP and also to the serine synthesis pathway (SSP) (see below) are important for the proliferation and survival of thyroid cancer cells.

In the in vitro experimental settings, growth of eight human thyroid cancer cell lines, including 8505C, K18, C643, TPC1, etc., is largely dependent on glucose [51]. Compared to Nthy-ori 3-1 cells, an immortalized normal human thyroid cell line, BCPAP and TPC1 cells are more glycolytic, as demonstrated by higher glucose uptake, LDH production and expression levels of GLUT1 and HK1/2, although their mitochondrial function is intact [31,52,53,54]. LDHA knockdown (KD) and overexpression decreases and increases the migration and invasion of these cells, respectively. Similarly, down-regulation of LDHA by an inducible KD approach decreases cell viability, clonogenic activity and tumor growth, and increases radio-sensitivity of an ATC cell line Hth83 [43]. MCT1 expression is positively correlated with proliferation/invasion/migration in TPC1 cells [28]. PKM2 is overexpressed in thyroid cancer cell lines of TPC1, K1 and BCPAP, which is due to aberrant down-regulation of the tumor suppressive miRNAs, miR-148a and miR-326 [42,52]. Decreased kinase activity of PKM2 compared to PKM1 facilitates diversion of glycolytic intermediates to biosynthetic pathways such as the PPP and the SSP (mentioned above) [55]. G6PD expression is higher in TPC1 than Nthy-ori 3-1 cells [52]. However, higher expression of PCB is also reported in TPC1 and 8505C cells vs. Nthy-ori 3-1 cells [27,56]; in these cancer cell lines, this enzyme plays a role in replenishment of the TCA cycle (entry of oxaloacetate into the TCA cycle) and increases OXPHOS and thereby positively controls cell division and invasion, suggesting that pyruvate is converted not only to lactate but also to oxaloacetate to maintain mitochondrial metabolism even in glycolytic thyroid cancer cells of 8505C and TPC1 [27]. Involvement of PCB in fatty acid synthesis is also proposed (see below).

The in vitro experiments also revealed that thyroid cancer cells with BRAF^V600E^ (BCPAP and 8505C cells) are more glycolytic than those without via HIF1α-MYC- peroxisome proliferator-activated receptor γ coactivator 1β (PGC1β) axis [29,57], and that glucose uptake is suppressed by vemurafenib, a BRAF inhibitor, in BCPAP cells having BRAF^V600E^ [29,54]. Of interest, Lee et al. have found localization of BRAF^V600E^ not only in the cytoplasm but also in mitochondria in PCCl3-BRAF^V600E^ cells. Mitochondrial BRAF^V600E^ suppresses apoptosis and OXPHOS, and these functions cannot be inhibited by tyrosine kinase inhibitors. This may be a molecular basis for limited efficacy of tyrosine kinase inhibitors [58]. The mutations of PTEN and TP53 are also reported to increase glucose uptake in WRO and FTC133 cell lines [59,60]. 

From the therapeutic perspective, the effects of glycolysis inhibition on cancer growth/survival have been reported: (i) a glycolysis inhibitor 2-deoxyglucose (2-DG; a HK inhibitor) inhibits proliferation of several thyroid cancer cell lines (8505C, BCPAP, TPC1, etc.) [51,52,57]. 2-DG also sensitizes cancer cells to chemotherapeutic agents (doxorubicin (DOX) and cisplatin), irradiation and sorafenib [51,57]. (ii) Another glycolysis inhibitor 3-bromopyruvate (an inhibitor for HK, GAPDH and MCT) combined with low glucose in an in vitro cell culture system and with ketogenic diet in an in vivo tumor model suppresses growth of 8505C, JL30 and BCPAP cells and 8505C tumors, respectively, the latter of which shows no apparent adverse effect on mice, but 3-bromopyruvate or ketogenic diet alone has no therapeutic effect in the in vivo model, indicating that their combination may be crucial [61]. Although it is not effective when used alone, the ketogenic diet consists of very low carbohydrate intake, thereby impairing glycolytic metabolism. Additionally, cancer cells cannot use ketone bodies efficiently, compared to normal cells [62]. (iii) F1,6-BP, by inhibiting glucose uptake through a negative feedback mechanism, suppresses cell proliferation and induces apoptosis in W3 cells [63]. (iv) Highly effective combination of a BRAF inhibitor vemurafenib (which induces a significant reduction in metabolic glucose demand) and a PDGF inhibitor imatinib (which increases metabolic energy demand) is also reported in BCPAP cells [54]. (v) LDHA inhibition by an inhibitor FX11 inhibits BCPAP tumor growth. This anti-tumor effect can be augmented when combined with autophagy inhibition by chloroquine, because LDHA inhibition induces protective autophagy [31]. Autophagy induced by chemotherapy, irradiation and a kinase inhibitor sorafenib is generally protective and pro-survival [64,65]. (vi) Inhibition of lactate export by inhibiting MCT4 with acliflavine, syrosingopine and AZD3965 significantly reduces proliferation of thyroid cancer cell lines such as 8505C, JL30 and TCO1 cells in a low glucose environment [66]. (vii) PKM2 KD by siRNA is also shown to suppress cell proliferation/colony formation and migration/invasion in TPC1 and K1 cells [42]. However, the efficiencies of the above inhibitors are far less than satisfactory, because inhibition of glycolysis alone has shown to lead to compensatory increases in OXPHOS and glutaminolysis [67]. 

The PPP inhibitors, such as G6PD inhibitors (6AN and DHEA) and transketolase inhibitors (oxythiamine and genistein), either individually or in combination (additively or slightly synergistically) suppress proliferation of BCPAP, K1, 8505C and SW1736 cells through apoptosis induction [49]. Dox-resistant 8505C and KAT-4 cells have higher G6PD expression, in which G6PD KD or a G6PD inhibitor physcion induces apoptosis and cancels resistance to Dox [68]. 

Because mitochondria are intact in most non-oncocytic thyroid cancers, glycolytic thyroid cancer cells are also sensitive to inhibitors for mitochondrial function and biosynthesis, such as atovaquone (a complex III inhibitor) [69], tigecycline (an inhibitor of mitochondrial translation) [70], mitotane (a cytochrome c inhibitor) [71] and metformin (an inhibitor of complex I, mitochondrial glycerophosphate dehydrogenase (MGPDH), fructose 1,6-bisphopsphate and glucose-6-phosphatase [72,73,74]. MGPDH, a critical enzyme for the glycerophosphate shuttle (see Figure 1), is overexpressed in thyroid cancers, and positively regulates cell growth and mitochondrial metabolism [74]. The effectiveness of metformin in treatment of thyroid cancer patients has also been reported [75]. Furthermore, combination therapies were attempted, showing the sensitization of thyroid cancer cells to paclitaxel, DOX and 2-DG by mitochondria inhibitors in 8505C, FTC133 BCPAP, TPC1, etc. [69,70,76]. These growth suppressive effects are no longer observed in thyroid cancer cell lines depleted with mitochondrial DNA (ρ0 cells), demonstrating that mitochondrial respiration is crucial for survival even in glycolytic thyroid cancer cell lines [69,70]. 

It is well known that ROS is a double-edged sword; ROS levels are generally higher in cancer cells which benefit their growth and migration, but when ROS increases more than a certain threshold, it induces cancer cell death. Moreover, the enhanced glycolysis drives ROS production [77] but at the same time ROS-scavenging molecules can be generated from the glycolytic intermediates through the PPP (see Section 2.1*. Physiology*). Thus, the role for ROS in cancer pathogenesis is complex. The same is true for thyroid cancer; numerous data show that anti-cancer effects of inhibition of glycolysis and also of mitochondrial function are at least in part attributed to ROS induction [49,63,70,78], and indeed that of F1,6-BP is cancelled by an antioxidant N-acetyl cysteine (NAC) [63]. On the other hand, however, the ketogenic diet reverses ROS levels and exerts a significant anti-cancer effect when combined with NAC in 8505C tumors [77]. 

In contrast, conflicting data are reported by Liu et al., which indicates that metabolic phenotype is the consequence, rather than the cause, of disease progression, and simply modifying the balance between glycolysis and OXPHOS would not be translated into beneficial effects in in vivo experimental settings (8505C, FTC133, etc.) [79]. 

Compared to non-oncocytic thyroid cancers, oncocytic thyroid cancers have dysfunctional mitochondria due to the loss-of-function mutations in mitochondrial DNA encoding the complex I and/or III of the ETC, essential for OXPHOS [5]. Metabolically, impaired OXPHOS makes the growth of oncocytic tumor cells totally dependent on glycolysis. Indeed, expression of glycolysis-related proteins such as GLUT1, HK II, carbonic anhydrase IX and MCT4 is higher in oncocytic cancers than non-oncocytic cancers [80], and FDG-PET signal is high, indicating high glucose uptake, in thyroid oncocytomas [81]. Thyroid oncocytic carcinoma cell line XTC.UC1 cells [82] exhibit higher glucose uptake than non-oncocytic cancer cells and cannot survive in glucose-free conditions [53,83,84]. However, LDH and lactate levels are not elevated or only marginally elevated in XTC.UC1 cells [85], presumably because glucose intermediate metabolites are used for synthesis of the glycosylation substrate UDP-GlcNAc in the HBP, for the SSP and for lipid metabolism [86,87]. Porcelli et al. have owed impaired glycolysis to the destabilization of HIF1α by higher αKG and normal succinate in XTC.UC1 cells [88]. Since HIF1α induces expression of genes responsible for glucose uptake (i.e., glucose transporters) and glucose breakdown, a lack of HIF1α expression inhibits glycolysis. However, Addie et al. found low aKG in the same cell line [86]. These data may represent heterogeneity within this cell line among different laboratories. In oncocytomas in other tissues, HIFα levels are variable; elevated HIF1α expression in benign renal oncocytoma and a human tumorigenic cell line C8T with heteroplasmic ND5 mutation [89,90,91] and comparable expression levels between pituitary oncocytomas and normal pituitary glands [92]. Shama et al. identified the pathway from complex I-defect to ROS elevation to AKT activation and to HIF1α elevation [90]. 

## 3. Amino Acid Metabolism

### 3.1. The Contents of Intracellular Amino Acids and Amino Acid Transporters

In general, cancer cells cannot adequately synthesize not only essential but also some non-essential amino acids, and thus have to overexpress amino acid transporters and uptake them from the extracellular tumor microenvironments to support rapid cell proliferation [93]. As a result, the concentrations of many amino acids are elevated in many cancer tissues [25,94]. This is also the case for thyroid cancers; several amino acid transporters such as SLC7A5 (LAT1), SLC1A5 (ASCT2) and SLC7A11 (xCT) are overexpressed in thyroid cancer tissues, with some of them correlating with patients’ poor prognosis [95,96,97]. Functionally a LAT1 inhibitor JPH203 or LAT1 KD by siRNA inhibits cell/tumor proliferation via mTOR in several thyroid cancer cell lines (8505C and OCUT-2/6) and in BRAF^V600E^;PIK3-CA^H1047R^ and 8505C tumors [96,97]. However, there is not a clear positive correlation between the suppressive effect and LAT1 expression [96].

### 3.2. The SSP and One-Carbon Metabolism

As mentioned above, glycolytic metabolic intermediates are used for biosynthetic product of macromolecules. This is evident in cancer cells. Thus, substantial amounts of 3PG generated in the glycolysis reaction is oxidized by phosphoglycerate dehydrogenase (PHGDH) to 3-phosphohydroxypyruvate, a precursor for de novo serine synthesis, which is then catalyzed by phosphoserine aminotransferase (PSAT1) and phosphoserine phosphatase (PSPH) to serine. Serine by itself changes to glycine and confers a methyl group to a one-carbon metabolism pathway by catalyzing with serine hydroxymethyltransferase (SHMT), which is important for nucleotide synthesis, methylation reaction, etc. [98]. 

In thyroid cancers, expression levels of enzymes for serine metabolism differ in distinct types of thyroid cancers; they are higher in PTCs than medullary cancers, higher in the conventional type than follicular variants, and higher in PTC with BRAF^V600E^ than those without [99], and higher in Hürthle oncocytic cell neoplasia than non-oncocytic follicular neoplasia [87]. In the metabolite analysis, higher levels of glucose but similar levels of 3PG suggest 3PG conversion to the SSP [100]. PHGDH KD by shRNA or functional inhibition by an inhibitor (NCT503) decreases, and its overexpression augments cell viability, colony formation and stemness phenotypes (spheroid formation and expression of embryonic cancer stemness markers, such as Oct4, Sox2, KLF4 and Nanog) in thyroid cancer cell lines 8505C and BCPAP, and normal Nthy-ori 3-1 cells [100]. There is positive feedback between serine and PKM2; serine activates PKM2 and PKM2 promotes the SSP. 

### 3.3. Glutamine Metabolism

Glutamine, its serum concentration being the highest among 20 amino acids, is one of the most important amino acids. Glutamine is taken up through a glutamine transporter ASCT2. Then, it typically refills the TCA cycle with α-ketoglutarate (αKG) through glutamine-glutamate-αKG pathway catalyzed by glutaminase (GLS) and glutamate dehydrogenase (GDH), finally producing GTP/ATP, NADPH and pyruvate. This pathway is called glutaminolysis (oxidative TCA cycle; the clockwise direction of the TCA cycle reaction in Figure 1)) [10,67,101]. Thus, glutamine drives the glucose-independent TCA cycle. Lower citrate but higher α-ketoglutarate in the metabolite analysis suggest glutamine influx into the oxidative TCA cycle [100]. Alternatively, α kg can also be used for generating citrate, which is then used for fatty acid synthesis (called reductive carboxylation; the counterclockwise direction of the TCA cycle reaction) [102], especially in the TCA cycle or ETC-defective cells [86,103,104]. Glutamine, together with cysteine and glycine, is also important for glutathione synthesis [86] and nucleotide synthesis as a nitrogen source [105]. 

In thyroid cancer tissues, expression levels of glutamine metabolism-related enzymes, GLS and GDH, are highest in AC followed by PTC, higher in PTC with BRAF^V600E^ than those without [106] and higher in Hürthle oncocytic cell tumors than non-oncocytic tumors [107]. Higher expression of these enzymes are markers for short survival and extrathyroidal extension [108]. In an oncocytic thyroid cancer cell line XTC.UC1, glutathione is used mainly for reductive carboxylation and glutathione synthesis and reductive carboxylation [86]. 

In non-oncocytic thyroid cancer cell lines (8505C, K18, C643, TPC1, etc.), their growth is dependent on glutamine, albeit to a lesser extent than glucose [51]. On the contrary, XTC.UC1 cells rely more on glutamine than TPC1 cells do (our unpublished data). Inhibition of GLS with its inhibitors (BPTES and CB-839) suppresses cell growth and migration in Ki, IHH4, BCPAP and TPC1 cells [108]. 

## 4. Lipid Metabolism

### 4.1. Physiology

Fatty acids are generated from a TCA cycle intermediate citrate in mitochondria or from acetate in the cytoplasm. Citrate in mitochondria is transported through the transporter protein citrate carrier out of mitochondria into the cytosol where it is cleaved by ATP-citrate lyase (ACLY) to generate acetyl-CoA. ACLY is also critical for histone acetylation (stimulating cell growth) and AMPK inhibition (preventing form cellular senescence). Acetate is converted to acetyl-CoA by ligation with CoA by acetyl-CoA synthetase (ACSS). Acetyl-CoA is then carboxylated by acetyl-CoA carboxylase (ACC) to generate malonyl-CoA, which is combined with acetyl-CoA to generate the 16-carbon saturated fatty acid (palmitate) by fatty acid synthase (FASN). Palmitate is then elongated by elongase (such as long-chain FA elongase also involved in cholesterol synthesis) and/or desaturated by desaturase (such as stearoyl-CoA desaturase (SCD)) to generate a variety of fatty acids. ACC is not only an enzyme involved in fatty acid synthesis, but also an inhibitor of carnitine palmitoyltransferase-I, a critical enzyme for fatty acid degradation (fatty acid oxidation or β-oxidation), and thus controls the balance between fatty acid synthesis and degradation. Expression of these enzymes is under the control of sterol regulatory element-binding protein (SREBP). Fatty acids synthesized are used to generate membrane molecules, such as glycolipids and phospholipids, signaling molecules, such as diacylglycerol (DAG) and phosphatidylinositol-3,4,5-triphosphate (PIP3), and energy storage molecules, such as triacylglycerides (TAG). 

Conversely, in β-oxidation, fatty acid is first converted to Acyl-CoA by acyl-CoA synthetase (or fatty acid-CoA ligase), transferred into mitochondria by carnitine palmitoyltransferase (CPT) and finally processed to acetyl-CoA by multiple enzymes including acyl-CoA dehydrogenase. 

### 4.2. Lipid Metabolism Reprogramming in Thyroid Cancers

Fatty acid synthesis is frequently increased in cancer cells to meet the above-mentioned requirements, but in contrast the significance of β-oxidation in cancer cells remains largely unknown. In thyroid cancers, the data on expression of enzymes in lipid metabolism are puzzling. FASN and/or stearoyl-CoA desaturase-1 (SCD1) is overexpressed [109,110,111], although their expression levels are not so high compared to cancers in other tissues [112]. SREBP expression is also high in thyroid cancers and associated with tumor sizes and lymph node metastasis [113]. Indeed, inhibition of FASN with C73 (a FASN inhibitor) suppresses AKT activation and cell growth and induces apoptosis in a BCPAP cell line [109], whereas stearoyl CoA desaturase 1 inhibitors (A939572 and MF-438) in ATC cell lines (THJ29T, THJ16T and KTC2), not PTC and FTC cell lines (TPC1, KTC1 and LAM1, EAM306, ML1, SDRA1 and FTC133) [114]. Higher expression of fatty acid synthesis enzymes through PCB (an enzyme involved in the anaplerotic flux into the TCA cycle by generating oxaloacetate from pyruvate (see above))-PI3K-Akt/mTOR-SREBP1c signaling pathway is also reported, and expression levels of PCB are positively correlated with thyroid cancer aggressiveness in patients with lymph node metastasis and in 8505C cells [56]. In contrast, ACC, especially ACC2, expression is down-regulated in PTCs with mBRAF compared to those without mBRAF and normal samples, facilitating fatty acid oxidation. BRAF inhibition increases ACC expression and lipid synthesis, and decreased β-oxidation in cancer cell lines (BCPAP, KTC1 and TPC1) [115]. Decreased ACCs is associated with vemurafenib resistance. Thus, it is suggested that ACC2 rescue may be a novel therapeutic choice [115]. Lu et al. have reported decreased TG and diacylglycerol and increased phospholipids/cholesterol in PTCs, and increased fatty acid metabolism-related enzymes (such as FA transport protein 2, acyl-coenzyme A dehydrogenase, CPT1A, lipoprotein lipase) in PTCs compared to normal counterparts in proteomics, indicating activated β-oxidation of fatty acid for energy production, conversion of fatty acid to phospholipids as cell membrane components and disintegration of fatty acid into secondary messengers for signal transduction pathways in TCs [116]. However, fatty acid synthesis and β-oxidation are mutually exclusive. 

In lipidomic (free fatty acid) profiling, 18 fatty acids, from C10 to C24, were higher in cancers than normal tissues, and tumor-specific lipids consisting of monounsaturated and saturated fatty acids were detected [117]. However, decreased levels of fatty acid esters are also reported in thyroid cancers compared to normal thyroids (with FFPE [24], and with fresh-frozen tissues [20,118]). Phosphatidylcholine (34:1), phosphatidic acid (36:3), and sphingomyelin (34:1) are reported to be useful for discriminating malignant from benign/normal [111]. Phosphatidylcholine (32:0, 32:1, 34:1 and 36:3), sphingomyelins (34:1 and 36:1) and phosphatidic acids (36:2 and 36:3) are higher in thyroid cancers than normal counterparts [119], and phosphatidylcholine (34:1 and 34:2) and sphingomyelin (34:1) are higher in PTC vs. normal tissues [120]. However, the significance of these unique fatty acid profiles in cancer is at present unclear. 

SREBP1, a transcription factor regulating lipogenesis, is also reported to be overexpressed in thyroid cancers, and to positively regulate cell proliferation and survival [113]. In addition, lipid composition can be useful for discriminating different types of thyroid cancers [24]. 

## 5. Conclusions

The agents used for metabolic reprogramming-targeted therapies attempted so far in thyroid cancer are summarized in Figure 1. Unfortunately, most remain as in vitro studies with cell lines. Since thyroid cancers utilize multiple pathways to drive rapid proliferation and resistance to cell death, each pathway can potentially be targeted, but at the same time inhibition of one pathway is likely to be compensated by the others. Furthermore, metabolic reprogramming-based therapies target molecules (mainly enzymes or transporters) common to both cancer and normal cells, although relative importance of each metabolic pathway differs between them, which makes adverse effects unavoidable and prevents progress to clinical trials. For example, the clinical trials of 2-DG were halted in other cancers because of limited efficacy and significant adverse effects [121]. Generally, a better therapeutic window can be obtained by targeting pathways that differ more between cancer and normal cells. 

Finally, patterns and degrees of metabolic reprogramming are likely diverse among different thyroid cancers, which hampers development of common therapeutic strategy for all thyroid cancers. Thus, more efforts will be necessary to scrutinize the metabolic profiling in individual thyroid cancer patients, and to identify thyroid cancer patients who would respond to such treatments.
